# A powerful transgenic tool for fate mapping and functional analysis of newly generated neurons

**DOI:** 10.1186/1471-2202-11-158

**Published:** 2010-12-31

**Authors:** Jingzhong Zhang, Florian Giesert, Karina Kloos, Daniela M Vogt Weisenhorn, Ludwig Aigner, Wolfgang Wurst, Sebastien Couillard-Despres

**Affiliations:** 1 Institute of Developmental Genetics, Helmholtz Zentrum Muenchen, German Research Center for Environmental Health (GmbH), Ingolstaedter Landstrasse 1, D-85764 Neuherberg, Germany; 2 Max Planck Institute of Psychiatry, Kraepelinstrasse 2-10, D-80804 Munich, Germany; 3 Institute of Molecular Regenerative Medicine, Paracelsus Medical University Salzburg, Strubergasse 21, 5020 Salzburg, Austria; 4 TUM, Technical University Munich, Chair of Developmental Genetics, c/o Ingolstaedter Landstrasse 1, D-85764 Neuherberg, Germany; 5 DZNE, Deutsches Zentrum für Neurodegenerative Erkrankungen, Ludwig Maximilian University, c/o Adolf Butenandt-Institut für Biochemie, Schillerstraße 44, 80336 Munich, Germany

## Abstract

**Background:**

Lack of appropriate tools and techniques to study fate and functional integration of newly generated neurons has so far hindered understanding of neurogenesis' relevance under physiological and pathological conditions. Current analyses are either dependent on mitotic labeling, for example BrdU-incorporation or retroviral infection, or on the detection of transient immature neuronal markers. Here, we report a transgenic mouse model (DCX-CreERT2) for time-resolved fate analysis of newly generated neurons. This model is based on the expression of a tamoxifen-inducible Cre recombinase under the control of a doublecortin (DCX) promoter, which is specific for immature neuronal cells in the CNS.

**Results:**

In the DCX-CreERT2 transgenic mice, expression of CreERT2 was restricted to DCX+ cells. In the CNS of transgenic embryos and adult DCX-CreERT2 mice, tamoxifen administration caused the transient translocation of CreERT2 to the nucleus, allowing for the recombination of loxP-flanked sequences. In our system, tamoxifen administration at E14.5 resulted in reporter gene activation throughout the developing CNS of transgenic embryos. In the adult CNS, neurogenic regions were the primary sites of tamoxifen-induced reporter gene activation. In addition, reporter expression could also be detected outside of neurogenic regions in cells physiologically expressing DCX (e.g. piriform cortex, corpus callosum, hypothalamus). Four weeks after recombination, the vast majority of reporter-expressing cells were found to co-express NeuN, revealing the neuronal fate of DCX+ cells upon maturation.

**Conclusions:**

This first validation demonstrates that our new DCX-CreERT2 transgenic mouse model constitutes a powerful tool to investigate neurogenesis, migration and their long-term fate of neuronal precursors. Moreover, it allows for a targeted activation or deletion of specific genes in neuronal precursors and will thereby contribute to unravel the molecular mechanisms controlling neurogenesis.

## Background

Neurogenesis is a strictly controlled process generating and maintaining the complex CNS cytoarchitecture. In the adult brain, neurogenesis constitutes in addition a form of cellular neuronal plasticity by continuously generating new neurons from resident neural stem cells (NSCs). Neurogenesis progresses through several sequential events, including proliferation, neuronal lineage restriction of precursors, cell cycle exit, migration and integration into target area, differentiation, as well as morphological and functional maturation. At the end of this process, newly generated cells can be found as functionally integrated and active neurons [1-3].

Neuronal precursors and newly generated neurons can be identified by their expression of doublecortin (DCX) [4,5]. In the adult CNS, expression of DCX is mainly detected in the adult dentate gyrus of the hippocampus and in the subventricular zone/rostral migration stream/olfactory bulb axis (SVZ/RMS/OB) [4,6-8]. Based on the close association between DCX expression and neurogenesis [5], we previously generated transgenic mice, to monitor neurogenesis *in vitro *and *in vivo*, in which reporter genes were driven by the DCX promoter [9-13].

The potential of involving adult neurogenesis in therapeutic strategies to replace pathological neuronal losses urges for a better understanding of neurogenesis at the molecular and cellular levels. In addition, accumulating evidence indicates that abnormal neurogenesis might be involved in the pathogenesis of neuropsychiatric disorders [14-16]. Therefore, to understand and dissect the molecular mechanisms driving neurogenesis *in vivo*, various models have been developed over the last years. For example, transgenic models have been generated based on cell-type specific promoters such as nestin, GLAST, PLP (proteolipid protein), or DCX to investigate the biology of neural stem cells, radial glia, oligodendroglial precursors and neuronal precursors, respectively [11,17-21]. However, these reporter mice are not suitable for long-term studies such as fate tracing or studies on the long-term functional integration of the newly generated neurons. For example, in the SVZ/OB axis and in the dentate gyrus DCX is expressed in newly generated neurons only transiently (mostly less than 1 month in rodents' DG and OB) [4], and thus, the DCX reporter mice are not applicable for fate mapping studies. In the other groups of mice, the GLAST or nestin promoter-driven expression of Cre-recombinase takes place in cells that are still multipotent and as a consequence the fate of these cells is not exclusively neuronal. Therefore, the lack of suitable models to study specifically neuronal precursors' long-term fate still constitutes a major deficit.

To remedy this absence of suitable tool for neuronal precursor fate analysis, we generated transgenic mice bearing the tamoxifen-inducible CreERT2 recombinase gene under the control of the DCX promoter. In this report, we demonstrate that this new transgenic tool allows for time-resolved permanent labeling of newly generated neurons and long-term analysis of their fate. Moreover, it provides a platform to induce and eliminate expression of genes in a crucial time window of neuronal maturation and study the functional consequences of these manipulations.

## Methods

### Plasmid Constructs

A 2380-bp *Sal *I-*Not *I fragment of pCAG-CreERT2-bpA-SS1 vector containing the CreERT2 cDNA was subcloned into the *BamH *I and *Not *I site of the phuDCX-3509-DsRed2 cassette [9], which contains the promoter region of human DCX, resulting in the phuDCX-3509-CreERT2 (Additional file 1). A 7.7-kb DCX-3'UTR (3'UTR) was amplified with RT-PCR, following the manufacturer's instructions (Invitrogen Kit; catalog No. 11904-018). PCR amplifications were performed with the sense primer 5'-*ACTAGT*AAGATGATAGGCTAAATCAAAGCC-3' and antisense primer 5'-*GCGGCCGC*TTTTTTTTTTTTTTTTTTTATTGAAATCAAATTTTAT-3'. The *Spe *I and *Not *I sites were inserted in the 5' terminal of primers respectively (the italic sequences with underlines). PCR products were cloned into a pCRII vector (TOPO TA Cloning Kit; Invitrogen; catalog No. K4600-01) to obtain the pCRII-TOPO-3'UTR plasmid. A 7.7-kb *Spe *I-*Not *I fragment of pCRII-TOPO-3'UTR was subcloned into the *Spe *I and *Not *I site of the phuDCX-3509-CreERT2 cassette to get the phuDCX-3509-CreERT2-3'UTR targeting plasmid.

### Generation of the DCX-CreERT2 transgenic mice

The targeting plasmid, phuDCX-3509-CreERT2-3'-UTR, was linearized by digestion with *Sal *I-*Not *I. The purified linearized DNA was microinjected into the pronuclei of fertilized oocytes of FVB inbred mice. Genotypes of the offspring were determined by PCR analysis and Southern Blot of tail DNA. An initial screen of the offspring was performed via PCR analysis with Cre sense primer 5'-TGCATTACCGGTCGATGCAAC-3' and the antisense primer 5'-GAAATCAGTGCGTTCGAACGCTAGA-3'. Cre-positive mice were further analyzed with Southern Blot (Additional file 1). A 1.27-kb *Sal *I-*Hind III *fragment of pCAG-CreERT2-bpA-SS1 vector was employed for preparing the probe with random primer (GE Healthcare Kit; catalog No. RPN 1633), the labeling probe was purified with MicroSpin S-300 HR (GE Healthcare Kit; catalog No. 27-5130-01) following the manufacturer's instructions. In case of positive insertion this probe detects a 7424 bp fragment after digestion of the genomic DNA with *Kpn I*, or two fragments after digestion with *EcoR V *(the size of one fragment is 8415 bp and the other at least is over 4103 bp). (All restriction enzymes are from Roche Applied Science).

### Transgenic Mice Treatments

Animal experiments were carried out in accordance with the Council of European Communities Directive of the 24 November 1986 (86/609/EEC) and approved by the HelmholtzZentrum Munich Institutional Animal Care and Use Committee. To expand the DCX-CreERT2 transgenic mouse line, DCX-CreERT2 transgenic mice were backcrossed with wildtype C57Bl/6J mice. DCX-CreERT2 transgenic mice were in addition mated with two reporter lines: CAG-CAT-EGFP mice [22] or ROSA26^lacZ ^mice [23] to obtain DCX-CreERT2:ROSA26^lacZ ^or DCX-CreERT2:CAG-CAT-EGFP double transgenic mice. Recombination activity in these lines induces expression of the corresponding reporter gene and was used for the various analyses described hereafter.

Tamoxifen (TAM, T-5648, Sigma-Aldrich) was dissolved in corn oil (C-8267, Sigma-Aldrich) at a stock concentration of 10 mg/ml. To analyze the expression of CreERT2 or reporter genes in embryonic stages 20 μg TAM/g bodyweight was injected once intraperitoneally (i.p.) into pregnant mothers at different gestational stages. One day after the TAM injection, embryos were dissected for whole-mount X-Gal staining or immunostaining. To follow the fate of cells targeted at embryonic stages during adulthood, the same protocol of TAM injection was employed in pregnant females of 17.5 day gestational stage, and the offspring was sacrificed at 2 months of age. To identify whether the CreERT2 recombination can be activated with a single TAM injection in adult brain (8 to 10 week-old), one dose of 200 μg TAM/g bodyweight was injected (i.p.) and mice were analyzed 1 day later. Finally, study of the expression patterns of CreERT2, reporter genes and cell-type specific markers in the adult brain was realized by injecting daily 100 μg TAM/g bodyweight (i.p.) for 5 consecutive days. These mice were analyzed 2 days (D2), 8 days (D8), 15 days (D15), or 29 days (D29) after the last TAM injection. For all experiment control mice were included that were injected with vehicle only, i.e. corn oil.

Twenty-four hours prior to perfusion, mice were injected with 200 μg/g bodyweight BrdU (5-Bromo-2'-deoxyuridine; B5002; Sigma-Aldrich) prepared in sterile PBS, pH7.4.

### Histology

For whole-mount X-gal staining, embryos were fixed by immersion in 4% paraformaldehyde (PFA), 5 mM EGTA, 10 mM MgCl_2 _in PBS solution for 30 minutes at room temperature (RT). They were then rinsed in 0.1 M sodium phosphate buffer pH 7.4 (PB), 2 mM MgCl2, 0.01% sodium deoxycholate, 0.02% NP-40, and incubated with X-gal staining buffer (0.1% X-gal, 2 mM MgCl_2_, 0.01% sodium deoxycholate, 0.02% NP-40, 5 mM K_3_Fe(CN)_6_, 5 mM K_4_Fe(CN)_6 _in PB) for several hours in the dark at 37°C to visualize the beta-galactosidase (β-gal) activity as a blue reaction product. Stained embryos were washed twice in PBS and post-fixed with 4% PFA in PBS overnight at 4°C. X-gal staining of free floating sections was carried out as described above, with the modification that sections were post-fixed with 4% PFA in PBS only 1 h at RT, and then lightly counterstained with Eosin Y (0.1%, E4382, Sigma-Aldrich).

Embryos for immunohistology were fixed by immersion in 4% paraformaldehyde (PFA) in 0.1 M phosphate buffer pH 7.5 for 2-8 hrs. Thereafter the whole embryo was embedded in paraffin, and sagittally sectioned (8 μm) using a Microm HM 355 s Microtome (Leica).

Brains of adult mice were removed after transcardial perfusion with 4% PFA in 0.1 M phosphate buffer pH 7.5. Brains were post-fixed for 2 hours in the same fixative. Thereafter, brains were immersed in 20% sucrose at 4°C overnight and embedded in OCT compound. Brains were sectioned using a Leica cryostat into serial coronal or sagittal sections (40 μm) for a systematic sampling of the entire brain.

### Immunostainings

For immunofluorescence staining, free-floating sections were rinsed with PBS and blocked with PBS++ (PBS++: 5% fetal calf serum, 0.3% Triton X-100 in PBS) for 1 h at RT. However, in cases of staining involving the detection of BrdU, sections were pretreated in 2 N HCl at 37°C for 30 min, followed by a 10 min neutralization in 0.1 M borate buffer and six washes in PBS prior to blocking. Then they were incubated in PBS++ containing primary antibody dilutions (see Table 1) for 24 hours at 4°C, followed by three 10 min washes in PBS. Sections were then incubated with secondary antibody conjugated to cyanine 2 (cy2), cy3 or cy5 diluted in PBS++ for 2 h at RT (1:400, Jackson ImmunoResearch Lab). After three 10 min washes in PBS, sections were incubated with 5 mg/ml 4',6'-diamidino-2-phenylindole (DAPI) (Sigma-Aldrich, D9564) for 20 min, followed by three 5 min washes in PBS. Finally, sections were mounted with Aqua poly/Mount (Polysciences, 18606).

**Table 1:** Primary antibodies used for immunohistology

Antigens	Antibodies	Dilutions	Companies
DCX	goat anti-DCX	1:200	Santa Cruz, sc-8066

choline acetyltransferase (CHAT)	goat anti-CHAT	1:100	Millipore, Chemicon, AB144P

Cre recombinase	rabbit anti-Cre	1:500	Abcam, ab24608

GFAP	rabbit anti-GFAP	1:1000	DAKO, Z0334

GAD65	rabbit anti-GAD65	1:1000	Millipore, Chemicon, AB5082

tyrosine hydroxylase (TH)	rabbit anti-TH	1:5000	Pel-Freez, P40101

calretinin	rabbit anti-calretinin	1:2000	Swant, 7699/4

ionized calcium-binding adaptor molecule 1 (Iba1)	rabbit anti-Iba1	1:500	Wako, 019-19741

GFP	chicken anti-GFP	1:1000	Aves, GFP1020

beta-galactosidase	chicken anti-beta-galactosidase	1:1000	Abcam, ab9361

NeuN	mouse anti-NeuN	1:200	Millipore, Chemicon, MAB377

calbindin D-28K	mouse anti-calbindin-D-28K	1:2000	Sigma-Aldrich, C9848

2', 3'-cyclic nucleotide 3'-phosphodiesterase (CNPase)	mouse anti-CNPase,	1:200	Millipore, Chemicon, MAB326R

NG2 chondroitin sulfate proteoglycan	mouse anti-NG2	1:500	Millipore, Upstate, 05-710

5-bromodeoxyurudine (BrdU)	rat anti-BrdU	1:500	AbD Serotec, OBT0030CX

vesicular glutamate transporter 2 (VGLUT2)	guinea pig anti-VGLUT2	1:5000	Millipore, Chemicon, AB5907

### Statistics

A minimum of 100 positive cells per region of interest in each animal and for each time point were counted to quantify the recombination events. Data are presented as mean ± SD.

## Results

### Generation of transgenic Dcx-CreERT2 mice

We previously demonstrated that a 3509-bp DCX genomic fragment could properly drive expression of reporter genes in neuronal precursors and immature neurons *in vitro *and *in vivo *[9,10]. Therefore, the CreERT2 encoding sequences were subcloned downstream of this DCX regulatory fragment (Additional file 1). Two male founders carrying the CreERT2 transgene were obtained after pronuclear injection. Both founders transmitted their transgene to the F1 generation and Southern blot analysis suggested that only 1 copy of the transgene was integrated into the host genome (Additional file 1).

Cre-recombinase activity was assessed on the F1 generation of both founder-derived lines following mating with Rosa26^lacZ ^reporter mice bearing a lacZ expression cassette activated following recombination [23]. Two month-old DCX-CreERT2:Rosa26^lacZ ^mice were perfused two weeks after a tamoxifen (TAM) or vehicle injection and stained for β-gal activity. Both DCX-CreERT2 transgenic lines exhibited the expected TAM-induced β-gal expression in the adult neurogenic regions, i.e. SVZ and dentate gyrus (Additional file 1). In contrast, no β-gal activity was observed following vehicle injections in the progeny derived from founder 2. However, in mice derived from founder 1, numerous β-gal positive profiles could be detected after vehicle injection indicating unspecific recombination events (data not shown). Therefore, only the transgenic DCX-CreERT2 founder 2 line was expanded and employed for the following experiments.

### Expression and sub-cellular localization of CreERT2 in DCX positive (DCX+) cells

**Figure 1:**
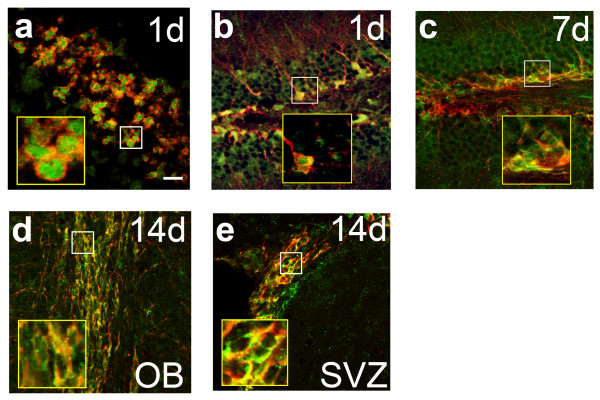
**CreERT2 cellular sub-localization in DCX-expressing cells**. a) Immunodetection showing subcellular localization of CreERT2 (green) within DCX-expressing cells (red) in the cortex of an E15.5 embryo injected with TAM at E14.5. b to e) Cre subcellular localization detected in the adult DCX-CreERT2 mice injected daily for 5 days with TAM. Immunodetection of CreERT2 (green) in the DG (b) 1 day and (c) 7 days after the last TAM injection in DCX-expressing cells (red). Immunodetection of CreERT2 (green) in the (d) OB and (e) SVZ 14 days after the last TAM injection in DCX-expressing cells (red). Insets show higher magnification of the selected areas. Scale bar in (a) = 33 μm

To validate that CreERT2 expression coincides with endogenous DCX expression in the DCX-CreERT2 transgenic mice, we investigated their respective expression patterns. At the cellular level, CreERT2 was detected in virtually all DCX+ cells of the developing CNS (E15.5). Furthermore, one day after injection of TAM, the CreERT2 has been translocated to the nucleus (Figure 1a). Similarly, in the adult brain, CreERT2 expression was, one day after TAM injection, strictly restricted to the nucleus of DCX+ cells (Figure 1b). Nuclear localization is induced by TAM administration and is a prerequisite for CreERT2 activity [24].

To determine the time window in which CreERT2 exerts its function in the nucleus after the TAM injection, DCX-CreERT2 adult mice were perfused at different time points post-injection and the sub-cellular localization of the CreERT2 was assessed. Seven days after TAM injection, the nuclear localization was dramatically decreased as compared to the first day. At this time point, CreERT2 expression was still co-localized with DCX, but its distribution returned to be mostly cytoplasmic (Figure 1c). Furthermore, two weeks after TAM injection, CreERT2 was exclusively localized in the cytoplasm (Figure 1d and 1e). Taken together, our results indicate that the CreERT2 nuclear localization rapidly recedes after the last TAM administration, indicating that CreERT2 activity was transient and virtually ceased after 7 days.

### Assessment of CreERT2 activity in neuronal precursor cells

Having confirmed the correct co-localization of CreERT2 with DCX+ cells, we then analyzed recombination activity and specificity. To this end, we mated DCX-CreERT2 mice with Rosa26^lacZ ^or CAG-CAT-EGFP reporter mice, which allow to monitor the activation of the respective reporter gene expression, following successful excision of the loxP-flanked cassette. The fate of DCX-expressing cells can then be followed by analyzing reporter gene expression at various time points following recombination.

**Figure 2:**
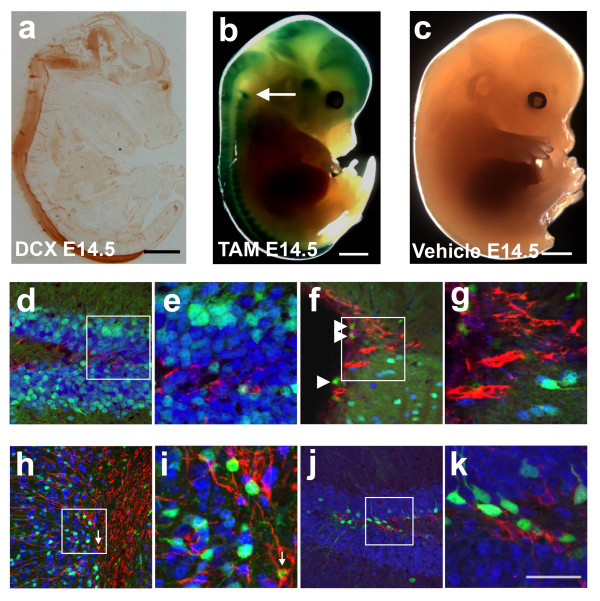
**Induction of reporter gene expression by TAM injections at different developmental stages**. a) Immunodetection of DCX expression in an E14.5 mouse embryo. Detection of β-gal activity at E15.5 in a DCX-CreERT2: Rosa26^lacZ ^embryos injected with (b) TAM or (c) vehicle at E14.5. Arrow in (b) points to a labeled dorsal root ganglion. No β-gal signal could be detected in vehicle-injected embryos. Immunodetection of DCX (red), NeuN (blue) and EGFP (green) in the DG (d and e) and SVZ (f and g) of a 2 month-old DCX-CreERT2:CAG-CAT-EGFP mouse injected with TAM at E17.5. Arrowheads in (f) point to EGFP-expressing cells remaining at the ependymal lining. Immunodetection of DCX (red), NeuN (blue) and EGFP (green) in the adult OB (h and i) and DG (j and k) of DCX-CreERT2:CAG-CAT-EGFP mice 4 weeks after TAM injection in the adulthood. Arrow in (i) points at the co-labeling EGFP with DCX at the anterior end of the RMS. Scale bars = 1.7 mm in (a), 2 mm (b) and (c), 50 μm (k).

The CreERT2 activity at embryonic stages was analyzed first. Twenty-four hours after a single TAM injection performed on E14.5, X-gal staining revealed that β-gal expression was restricted to the developing CNS and dorsal root ganglia (DRGs) (Figure 2). This distribution coincided with the pattern of endogenous DCX expression at this stage (Figure 2a). Activation of CreERT2 at E17.5 by a single TAM-injection led to a wide distribution of EGFP reporter expression in the adult brain (Figure 2d to 2k and Additional file 2). EGFP+ cells were detected in most regions of the brain parenchyma, such as in the granular cell layer of dentate gyrus, striatum, cortex, thalamus, Ammon's horn (CA1), etc. according to DCX expression pattern at E17.5.

Noteworthy, virtually all EGFP+ cells expressed NeuN, and thus had become mature neurons (Figure 2d to 2g). EGFP expression was neither detected in DCX-positive cells in the SVZ (Figure 2f and 2g), nor in the rostral migrating stream (RMS) (data not shown) and subgranular zone (SGZ) of the dentate gyrus (Figure 2d and 2e). A few EGFP+ cells could be found in or in close association to the ependymal layer of the lateral ventricles (Figure 2f, arrows). These EGFP+ cells, however, did neither co-express NeuN nor DCX and their nature remains to be elucidated.

Due to the lower numbers of neurons continuously generated in the adult CNS, adult mice were injected with TAM on 5 consecutive days and then perfused for analysis 4 weeks after the last injection. At this time point, SVZ-generated EGFP+ cells reached the OB and were distributed mainly within the granular cell layer (GrO) (Figure 2h and 2i) and to a lower extent in the periglomerular cell layer (pGl). The EGFP+ cells present in the OB were found to express the mature neuronal marker NeuN, whereas no co-expression of DCX could be detected (Figure 2h and 2i). Within the rostral RMS in contrast, a few scattered EGFP-expressing cells appeared to have retained expression of DCX. Moreover, only rare EGFP+ cells could be found in the SVZ (data not shown). Similarly to cells detected in the OB, four weeks after TAM administration, EGFP+ cells in the dentate gyrus expressed NeuN and were devoid of DCX (Figure 2j and 2k).

**Figure 3:**
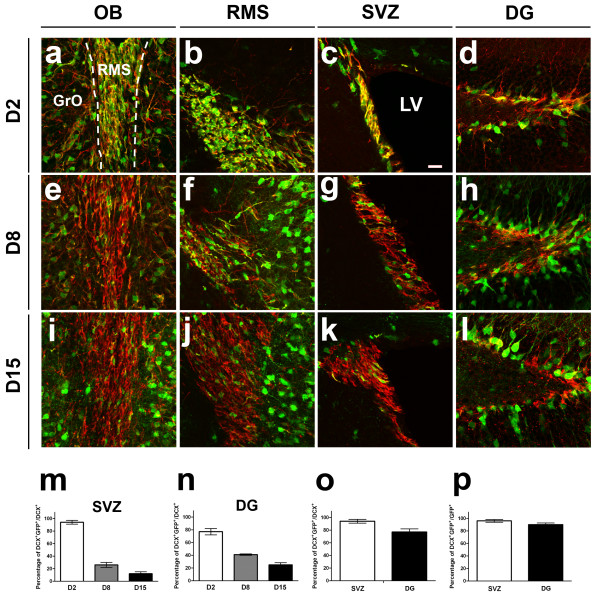
**Co-localization of EGFP and DCX expression following TAM administration**. Immunodetection of DCX (red) and EGFP (green) in the neurogenic regions of adult DCX-CreERT2:CAG-CAT-EGFP mice 2 days (a-d), 8 days (e-h) and 15 days (i-l) following last TAM administration. The extensive co-localization of DCX and EGFP observed at D2 rapidly decrease over time. The frequency of co-localization between EGFP and DCX was quantified at various time points. Percentages of EGFP-labeled cells among all DCX-expressing cells decreased over time in the SVZ (m) and in the DG (n). The efficiency of recombination, i.e. the percentages of all DCX+ cells that expressed EGFP, in shown in (o), whereas the specificity, i.e. the percentages of all EGFP+ cells expressing DCX is shown in (p). LV: lateral ventricle. Scale bar in (c) = 33 μm

The efficiency and the specificity of the recombination event in DCX-expressing cells were evaluated in the adult SVZ and SGZ. The percentage of all DCX+ cells expressing EGFP was defined as the efficiency of recombination, and the percentage of all EGFP+ cells expressing DCX was defined as the specificity. Since levels of accumulated EGFP within cells were relatively low at the earliest time point studied, EGFP signals were amplified using an anti-EGFP antibody in all following experiments. In the SVZ, we observed that approximately 94% DCX+ cells were co-expressing EGFP two days after the last TAM injection (Figure 3c and 3o). At the same time point in the SGZ of the dentate gyrus, EGFP could be detected in approximately 77% of DCX-expressing cells (Figure 3d and 3o). Moreover, approximately 96% EGFP+ cells in SVZ and 90% EGFP+ cells in SGZ (Figure 3p) were co-expressing DCX, demonstrating that the CreERT2 activity was highly efficient and specific.

In order to characterize further the kinetics of the DCX+ cells' emigration from their site of birth to their target structures, the distribution of EGFP-expressing cells following recombination was investigated over time. To this end, adult mice were sacrificed eight, fifteen or twenty-nine days after the last TAM injection (D8, D15 and D29, respectively). Co-localization of EGFP expression with DCX and NeuN was analyzed within the SVZ-RMS-OB axis and within the dentate gyrus at these time points.

In contrast to the high percentage of EGFP-expression within DCX+ cells two days after the last TAM injection, the percentage of EGFP-expressing DCX+ neurons in the SGZ decreased to 41% at D8, and declined gradually to roughly 25% of all DCX+ cells at D15 (Figure 3). Concomitantly, the frequency of co-localization in the SVZ decreased at D8 to 26.7%, further diminished to 12.5% at D15 (Figure 3). Finally, at D29, only rare EGFP+ cells remained within the SVZ and were found to express DCX, whereas no co-localization could be detected in the SGZ at this time point. Taken together, our data indicate that the main emigration wave of EGFP-labeled neurons departed away from the SVZ within the first 15 days (Figure 3).

### Neuronal phenotypes of EGFP+ cells integrated into adult neurogenic regions

**Figure 4:**
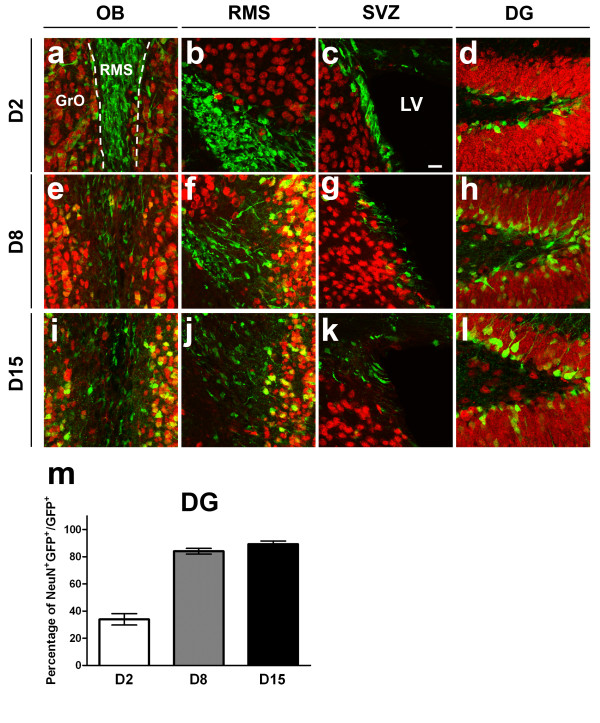
**Co-localization of EGFP and NeuN expression following TAM administration**. Immunodetection of NeuN (red) and EGFP (green) in the neurogenic regions of adult DCX-CreERT2:CAG-CAT-EGFP mice 2 days (a-d), 8 days (e-h) and 15 days (i-l) following last TAM administration. The frequency of co-localization between EGFP and NeuN increased over time and was quantified in the DG (m). LV: lateral ventricle. Scale bar in (c) = 33 μm

EGFP+ cells migrating along the RMS from D2 to D15 were found to maintain an immature neuronal morphology and only rare co-localization with NeuN could be documented (Figure 4). Over the next 4 weeks, expression of NeuN within EGFP+ cells was broadly induced as cells reached the GrO or the pGl of the olfactory bulb (Figure 2h and 2i, Figure 4 a, e and 4i). Notably, in the GrO, we found only weak expression of DCX in EGFP+ cells. In contrast, DCX was still strongly expressed in the cytoplasm of EGFP+ located at the anterior end of the RMS (Figure 2i, arrow), revealing that the expression of DCX decreased gradually as EGFP+ cells migrated into their target areas (data not shown). These observations reveal that DCX expression in cells migrating to the olfactory bulb is regionally regulated. In a similar fashion, EGFP-expressing cells in the dentate gyrus integrated in the inner granular layer over time and gradually induced the expression of NeuN. Quantitative analyses revealed that 15 days after the last TAM administration, more than 80% of EGFP+ cells found in the dentate gyrus (Figure 4m) and virtually all EGFP+ cells found in the olfactory bulb (data not shown) expressed the mature neuronal marker NeuN.

**Figure 5:**
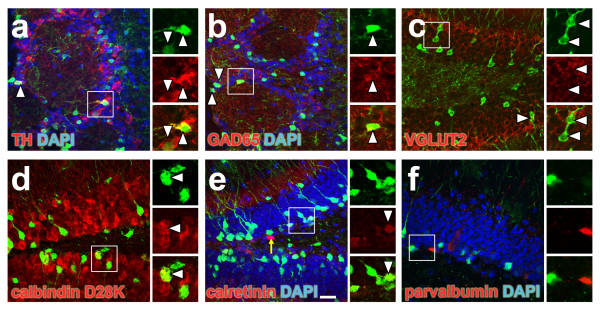
**Co-localization of EGFP and neuronal subtype-specific markers**. Twenty-nine days after the last TAM administration, the EGFP-expressing cells (green) were examined for the expression of specific markers (red). (a) co-localization with the dopaminergic marker tyrosine hydroxylase (TH) in the pGI, (b) co-localization with the GABAergic marker glutamate decarboxylase 65 (GAD65) in the pGI and (c) co-localization with the glutamatergic marker vesicular glutamate transporter 2 (VGLUT2) in the DG. (d) Co-localization with DG mature granule cell marker calbindin-D28K, (e) co-localization with the early DG granule cell marker calretinin. Arrow points to a cell expressing high levels of calretinin, but no EGFP, (f) absence of co-localization with parvalbumin in the DG. Scale bar in (e) = 33 μm.

To further characterize the neuronal phenotypes of EGFP-labeled neurons, the presence of neurotransmitter-specific markers and calcium-binding proteins was investigated by immunohistology at D29 (Figure 5). In agreement with previous studies [25,26], expression of GAD65, a marker found in GABAergic neurons, could be detected at this time point in EGFP-expressing cells located in the OB. Moreover, a sub-population of periglomerular EGFP+ cells was found to co-express TH, a marker specific for dopaminergic neurons (Figure 5).

On the other side, VGLUT2, a commonly used marker for glutamatergic terminations, could be detected in the granular layer of the dentate gyrus and surrounded EGFP+ cells at D29, revealing that EGFP-expressing cells received glutamatergic inputs (Figure 5). In addition, we scrutinized for the expression of the calcium-binding proteins calbindin-D28K, calretinin and parvalbumin in the EGFP-labeled granule neurons at this time point. The calbindin-D28K, which is expressed in mature granule neurons, could be detected in most EGFP+ cells of the dentate gyrus (Figure 5). In contrast, no parvalbumin and only weak expression of calretinin could be detected in EGFP+ cells of the dentate gyrus at this time point, although cells expressing high levels of parvalbumin or calretinin, the latter been specifically found in newly generated granule cells, could be detected in the vicinity (see for example arrow in Figure 5e).

### Proliferative capacity of EGFP+ cells in the adult neurogenic regions

**Figure 6:**
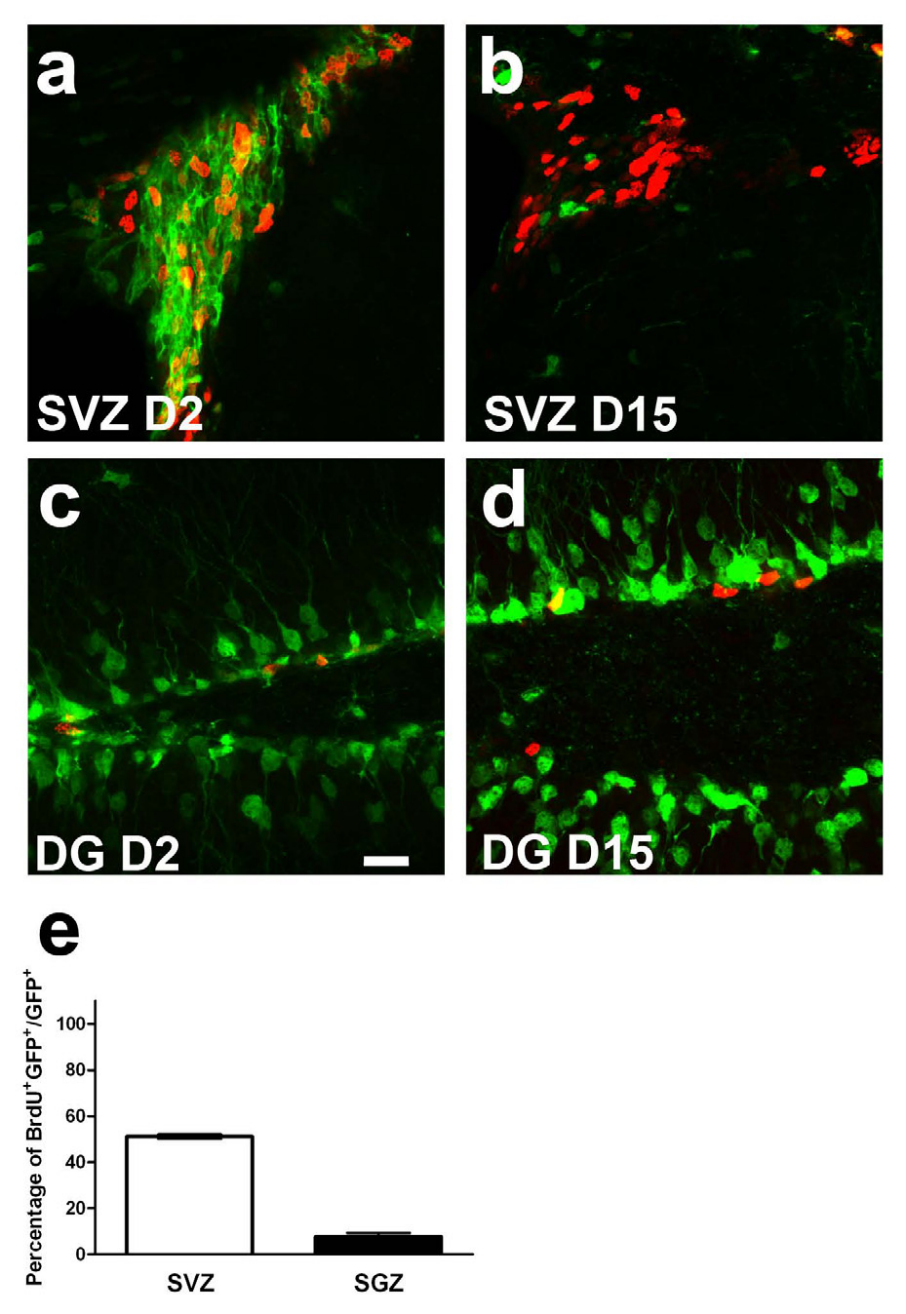
**Proliferative potential of EGFP-labeled cells**. One day or 14 days after the last TAM injection, dividing cells were labeled via a single BrdU injection and co-localization of EGFP with BrdU was examined at day 2 (a and c) and day 15 (b and d), respectively, in the SVZ and DG. Quantification on day 2 revealed that the percentage of all EGFP+ cells labeled with BrdU rapidly decreases and is shown in (e). Scale bar in (c) = 33 μm.

DCX expression takes place in a relatively heterogeneous population of neuronal precursors and young neurons of various maturation stages and proliferative statuses. Administration of BrdU to label proliferative cells was performed at various time points following recombination. At the earliest time point investigated, D2, approximately 51.1% of EGFP+ cells in the SVZ, but only 7.7% of EGFP+ cells in the SGZ incorporated BrdU respectively, confirming that a fraction of the cells was still proliferating (Figure 6). The higher proportion of mitotically active cells in the SVZ might be explained by the emigration of post-mitotic young neurons out of the SVZ towards the OB, leaving the most immature cells behind. Furthermore, no more BrdU incorporation in EGFP+ cells was observed in SGZ at D15 (Figure 6), underscoring the temporally limited proliferative capacity of DCX+ cells. In contrast, although no more BrdU incorporation in EGFP+ cells was observed in SGZ at D15, a few BrdU+/EGFP+ co-labeling cells could be found in RMS (data not shown), suggesting that some EGFP+ cells in SVZ/RMS/OB axis could keep proliferative capacity until at least D15.

### EGFP+ cells outside of adult neurogenic regions

Following administration of TAM to adult DCX-CreERT2:CAG-CAT-EGFP mice, EGFP+ cells could be detected outside of the described neurogenic regions. In this respect, scattered DCX-expressing cells have previously been reported in adult cerebral cortex of rodents, cats and primates [7,27]. To validate that EGFP expression in cells located outside of the neurogenic regions ensued from concomitant expression of DCX, we scrutinized by immunohistochemistry DCX expression pattern in the whole adult brain in respect to the activation of the EGFP expression.

**Figure 7:**
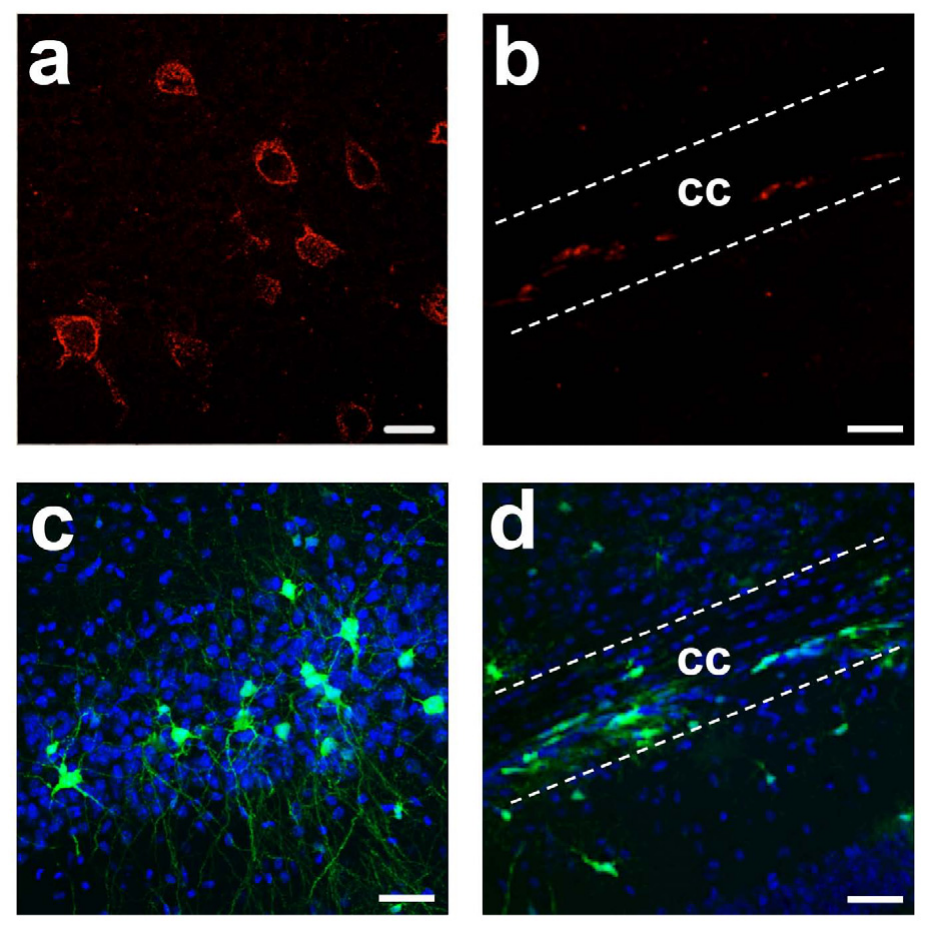
**Expression of DCX and EGFP outside of neurogenic areas**. Low levels of DCX expression can be detected outside of neurogenic areas. For example, DCX-expressing cells were detected by immunohistochemistry in the piriform cortex (a) and in the corpus callosum (b). Accordingly, EGFP-expressing cells were detected following TAM administration in the piriform cortex (c) and corpus callosum (d). Scale bar in (a) = 33 μm; Scale bar (b), (c) and (d) = 50 μm.

Low to moderate levels of DCX expression could be detected in cells dispersed throughout the cerebral cortex (Figure 7). Some weak DCX expression could also be perceived in corpus callosum, as well as around the 3^rd ^ventricle and hypothalamus, the molecular cell layer (MCL) and the granular cell layer (GCL) of cerebellum (data not show). Four weeks after TAM injection, EGFP+ cells also appeared in these regions (Figure 7). The reporter expression levels however were significantly higher than the endogenous DCX expression levels, since upon recombination, expression of the reporter gene was under the control of a strong promoter. Importantly, EGFP+ cells located outside of the neurogenic regions were not proliferative, as demonstrated by the absence of BrdU incorporation. Therefore, the nature and fate of these immature neurons remains to be deciphered.

## Discussion

In this report, we demonstrated that CreERT2-mediated recombination can be efficiently and specifically targeted *in vivo *in DCX-expressing cells, i.e. in the neuronal precursors and young neurons, using a DCX promoter-driven CreERT2. In contrast to DCX promoter-driven reporter lines previously generated [10,11], the DCX-CreERT2 construct described here additionally encodes the 3'UTR region of the DCX mRNA. The large DCX 3'UTR is known to contain post-transcriptional regulation elements of gene expression [28]. However, under experimental conditions used in this study, no overt differences in the expression pattern of the CreERT2 transgene could be documented in comparison to former lines devoid of DCX 3'UTR sequences. Nevertheless, the presence of DCX 3'UTR could lead to a more faithful CreERT2 expression pattern within the DCX-expressing cell population and more investigation will be required to understand its function.

One day after TAM administration, the CreERT2 protein was translocated to the nuclear compartment of DCX-expressing cells where it could proceed to recombination. The latter resulted in a rapid activation of reporter expression; β-gal or EGFP could be readily detected in the embryonic, as well as in the adult CNS, one day after tamoxifen injection (Figure 2). Following five daily tamoxifen administrations, 94% DCX+ cells in SVZ and 77% DCX+ cells in the dentate gyrus induced expression of EGFP, demonstrating a high efficiency of recombination using our experimental paradigms. On the other side, about 96% of EGFP+ cells in SVZ and 90% of EGFP+ cells in SGZ were co-expressing DCX, confirming that the recombination activity was induced with high specificity. (Figure 3). As the fate of EGFP+ cells was observed to be solely neuronal, we conclude that the absence of DCX expression in a small fraction of the EGFP+ resulted from the maturation-associated downregulation of DCX+ over the 5 days of TAM injection.

Although the nuclear translocation of the CreERT2 was transient, expression of the reporter got permanently induced following recombination, which allows for the long-term analysis of cell types arising from DCX+ cells. Hence, one month after recombination in the DCX-CreERT2, the vast majority of the EGFP+ cells in the neurogenic target regions expressed NeuN, a marker found in mature neurons. In addition, a few EGFP+ cells along the neuroblasts' migratory route or localized outside neurogenic regions were found to express low levels of DCX. Importantly, one month after the last TAM injection, no co-localization between the EGFP signal and the expression of GFAP (astrocyte marker), CNPase (oligodendrocyte marker) or Iba I (microglia marker) was detected (data not shown). This substantiates evidences that DCX-expressing cells are determined to become neurons under physiological conditions. This contrast with the use of the nestin promoter to drive the expression of the CreERT, which resulted in the labeling of the neural stem cell population generating thereafter a continuous flow of new neurons and glia [17].

Calbindin-D28K, calretinin and parvalbumin belong to the superfamily of low molecular weight calcium-binding proteins and are characteristic of different subpopulations of neurons [29]. Hence, in the dentate gyrus, Calbindin-D28K is as marker of mature granule cells while calretinin is transiently expressed in postmitotic newly generated neurons [30,31]. In agreement with the latter, we observed that EGFP+ granular cells in the dentate gyrus were expressing Calbindin-D28K, a few of them expressed low levels of calretinin, and none of them expressed parvalbumin four weeks after recombination (Figure 5). The absence of co-localization between EGFP and parvalbumin reinforced evidence that this GABAergic subpopulation is not replenished by a constant addition of new neurons in adult dentate gyrus, although this matter is still under debate [32-34].

### The EGFP+ cells outside the classical adult neurogenic regions

Following recombination in DCX-CreERT mice, EGFP+ cells were also found within non-neurogenic brain areas. In mammals, DCX+ cells have been reported outside neurogenic regions, including temporal and prefrontal cortex layer II, piriform cortex layer III/endopiriform nucleus, corpus callosum, nucleus accumbens, ventromedial striatum, ventrolateral septum, bed nucleus of the stria terminalis, molecular cell layer, granular cell layer and white matter of cerebellum. The distribution and frequency of these DCX+ cells appear to increase in superior species of the phylogeny, although it remains to be elucidated to which extent this is due to an increase of DCX expression levels and thereby a better immunohistochemical detectability [7,8,35-37].

Intriguingly, the numbers of EGFP+ cells around the 3^rd ^ventricle and hypothalamus appeared markedly higher than the numbers of DCX+ cells detected in these regions. The reason of this discrepancy may be related to a very low expression level of DCX in these cells, which would be hardly detectable using current antibodies. In contrast, once DCX-associated recombination occurred, the expression of reporter genes is controlled by a strong constitutive promoter allowing for an easy detection of targeted cells.

The origin and function of DCX-expressing cells outside of the neurogenic regions remain to be elucidated. For instance, it was reported that the neural stem cells can be isolated from virtually every region of the adult CNS [38]. In addition, upon specific treatment, neurogenic events have been induced within the adult cortex, striatum, CA1 region of the hippocampus, and even within the white matter [39-42]. It is tempting to conclude that some of the DCX-expressing cells detected in these regions were generated by a very low rate continuous neurogenesis. However, the absence of BrdU labeling obtained in these cells under physiological conditions suggests that such a mechanism of DCX+ cells generation would be marginal in the best case.

On the other side, there exists good evidence that the extra-neurogenic DCX-expressing cells might have been generated during developmental neurogenesis, but never fully completed their maturation process [43]. These cells would remain in the parenchyma as "quiescent" local neuronal precursors. The existence of such quiescent precursors has also been suggested following grafting experiments in which neural stem cells were injected into the ventricles during cortical development [44]. Detection of these cells at a later time point revealed that stem cells were integrated as neurons into various brain regions, but at the same time, a certain amount of cells remained as immature neurons in the parenchyma, potentially as a reservoir of precursor cells available for plasticity or local repair.

Recently, another mouse model expressing the DCX-CreERT has been reported [34]. In contrast to our model based on the human DCX promoter, Cheng and colleagues used a BAC construct encoding the murine DCX promoter. The latter, together with a possible positional effect of the transgene may explain the differences observed between the two models. For instance, in contrast to our mouse model, the model from Cheng et al. is exclusively active in DCX-expressing cells within the hippocampus [34]. Also, the authors claim that recombination in their DCX-CreERT mice takes only place in post-mitotic neuronal precursors. Given the fact that a significant fraction of all DCX-expressing cells are still in a proliferative state (see Figure 6 and [10]), this suggests that the induction of the DCX-CreERT transgene expression reported by Cheng is delayed in respect to the endogenous DCX. Thus, the DCX-CreERT mouse model presented by Cheng and colleagues appears to be well-suited for the study addressing the maturation and fate of newly generated granule cells of the dentate gyrus. Still, there is a lack of model(s) addressing the fate of DCX-expressing cells located outside of the dentate gyrus - such as in the subventricular zone (SVZ) - which is now possible by the model presented in this manuscript.

## Conclusion

We report here a transgenic mouse model based on an inducible Cre recombinase driven by the DCX promoter (DCX-CreERT2). Taken the high specificity and efficiency of recombination in neuroblasts and young neurons, this transgenic mouse model constitutes a powerful tool for tracing neurogenesis and fate-analysis of newly generated neurons. It is moreover a valuable model for studying molecular mechanisms of neural plasticity and neurogenesis through induction or silencing of specific genes in neuroblasts and young neurons. Finally, the possibility to analyze the long-term fate of newly generated neurons will be an asset in the development of innovative therapies against neurologic diseases.

## Authors' contributions

JZ: the main author, participated in the design of the study, performed all associated mouse breeding and histological work, did most of the molecular work and all histological analysis and drafted the manuscript. FG: Performed some essential clonings and interpreted together with JZ the molecular work. KK: assisted in the analysis of the histological sections. DMVW (responsible for the project coordination), LA, WW and SCD were initiating and designing the study; reviewed (DMVW, LA, WW) and wrote the manuscript (SCD). All authors contributed to the project with continuous updates on the research topic and influenced thereby the progress of the project. All authors have reviewed and contributed to the different draft versions of the manuscript. All have read and approved the final manuscript.

## Supplementary Material

Additional file 1 **The DCX-CreERT2 construct and the generation of DCX-CreERT2 transgenic mouse**. (a) Schematic representation of the DCX-CreERT2 construct. The 7.7-kb fragment DNA of Dcx-3'-UTR was cloned by RT-PCR and inserted downstream of the CreERT2. The founders were genotyped by (b) PCR and (c) Southern blot (non-transgenic control DNA in lane "C"). Two weeks after TAM injection, brains of adult mice originating from founder-derived lines mated with Rosa26^lacZ ^mouse (DCX-CreERT2:Rosa26) were further analyzed by X-gal staining. (d) β-gal positive cells in the DG of an adult DCX-CreERT2:Rosa26 mouse two weeks after TAM injection. (e) Absence of β-gal activity in the DG of a DCX-CreERT2:Rosa26 mouse injected two weeks before with the vehicle only (corn oil). Insets show higher magnification of the selected region. E, *EcoR V*; K, *Kpn I*. Scale bar in (e) = 50 μm. Click here for file

Additional file 2 **EGFP expression in the adult brain of DCX-CreERT2:CAG-CAT-EGFP mice injected once with TAM at E17.5**. Numerous EGFP-expressing cells could be detected, for example, (a) in the GrO, (b) layer II and VI of cerebral cortex, (c) striatum, (d) hippocampal pyramidal layer CA1 (e) and Purkinje cell of cerebellum. gl, glomerular cell layer; Mi, mitral cell layer of olfactory bulb; GrO, granular cell layer of olfactory bulb; Py, pyramidal cell layer of the hippocampus; Rad, stratum radiatum of the hippocampus; MCL, molecular cell layer of cerebellum; GCL, granular cell layer of cerebellum. Scale bar in (e) = 50 μm. Click here for file
