# Supplementary figures and images for: A powerful transgenic tool for fate mapping and functional analysis of newly generated neurons
Source: BMC Neurosci. 2010 Dec 31;11:158. doi: 10.1186/1471-2202-11-158 (PMC3019205; doi:10.1186/1471-2202-11-158)

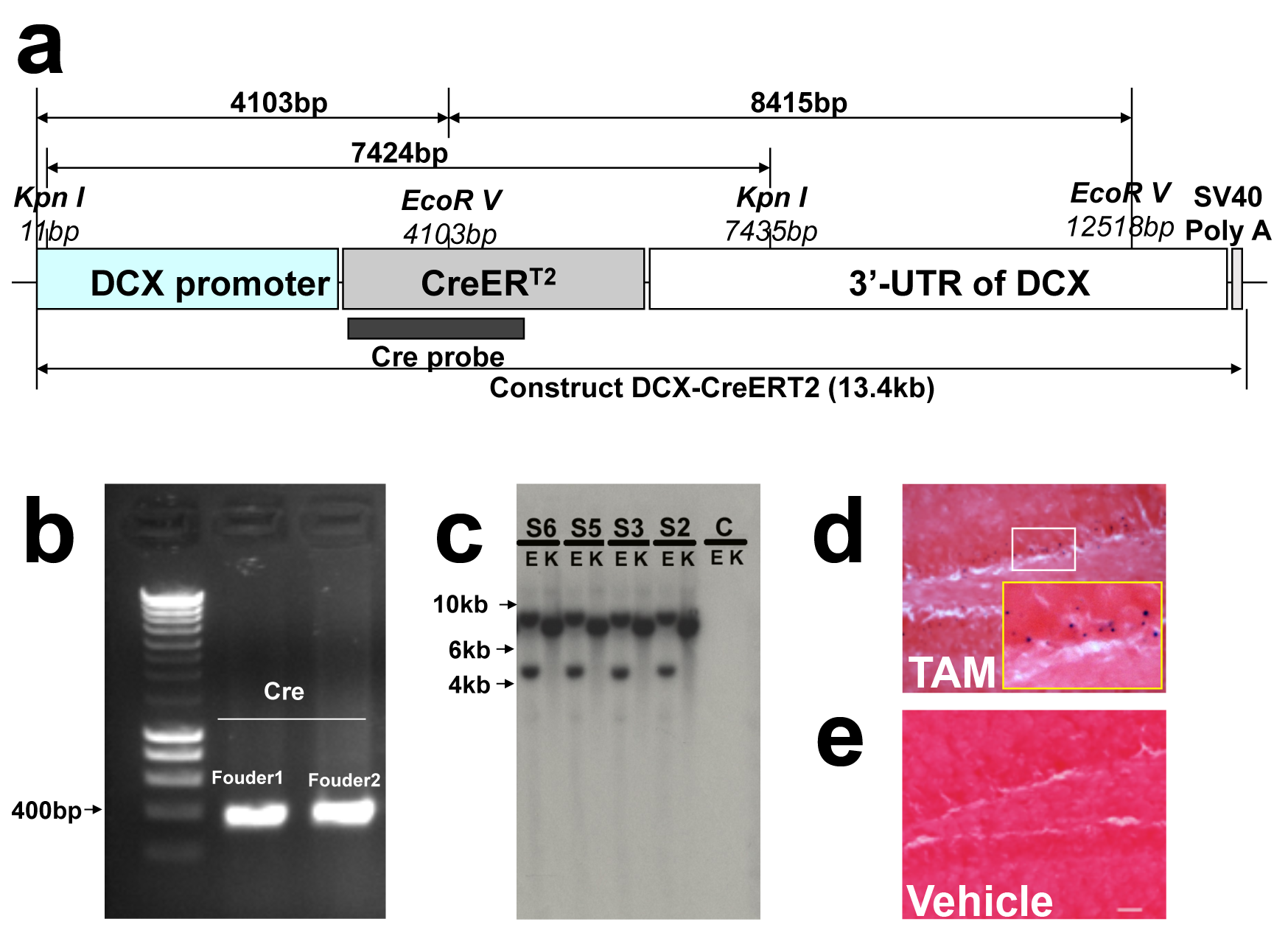

Supplement: Additional file 1 — The DCX-CreERT2 construct and the generation of DCX-CreERT2 transgenic mouse. (a) Schematic representation of the DCX-CreERT2 construct. The 7.7-kb fragment DNA of Dcx-3'-UTR was cloned by RT-PCR and inserted downstream of the CreERT2. The founders were genotyped by (b) PCR and (c) Southern blot (non-transgenic control DNA in lane "C"). Two weeks after TAM injection, brains of adult mice originating from founder-derived lines mated with Rosa26lacZ mouse (DCX-CreERT2:Rosa26) were further analyzed by X-gal staining. (d) β-gal positive cells in the DG of an adult DCX-CreERT2:Rosa26 mouse two weeks after TAM injection. (e) Absence of β-gal activity in the DG of a DCX-CreERT2:Rosa26 mouse injected two weeks before with the vehicle only (corn oil). Insets show higher magnification of the selected region. E, EcoR V; K, Kpn I. Scale bar in (e) = 50 μm. [file 1471-2202-11-158-S1.TIFF]

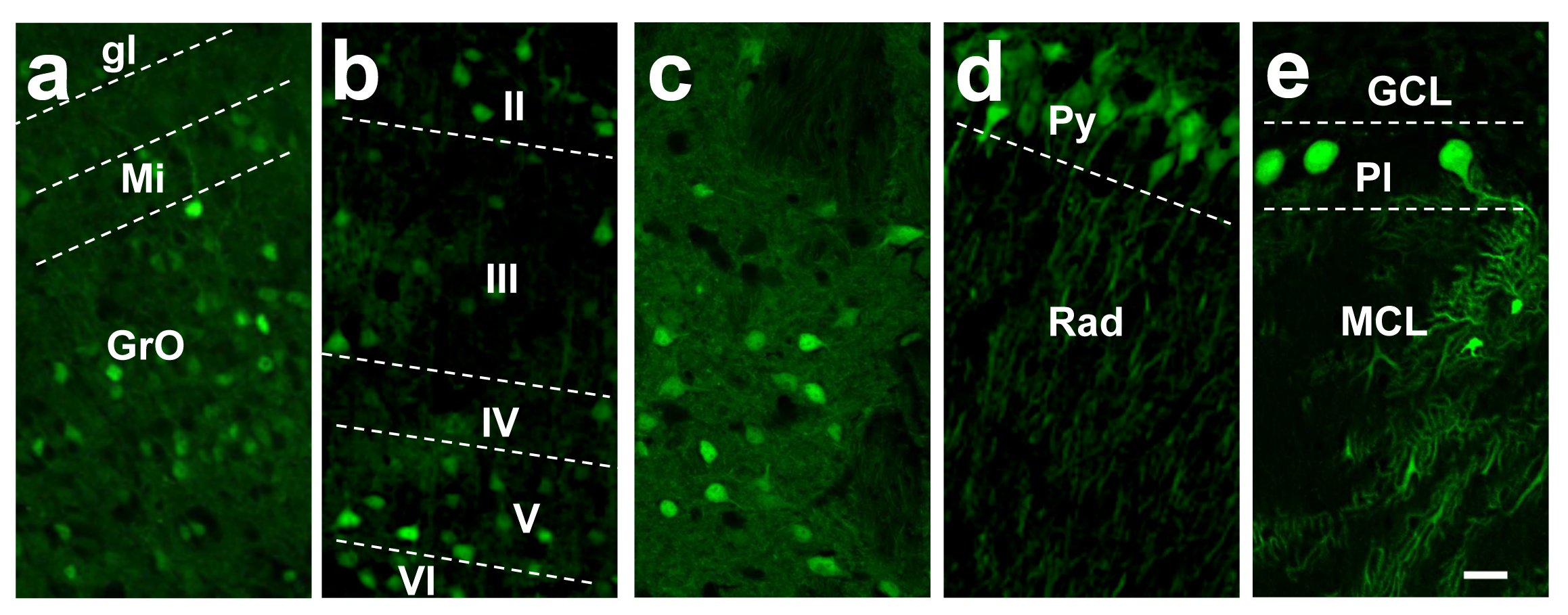

Supplement: Additional file 2 — EGFP expression in the adult brain of DCX-CreERT2:CAG-CAT-EGFP mice injected once with TAM at E17.5. Numerous EGFP-expressing cells could be detected, for example, (a) in the GrO, (b) layer II and VI of cerebral cortex, (c) striatum, (d) hippocampal pyramidal layer CA1 (e) and Purkinje cell of cerebellum. gl, glomerular cell layer; Mi, mitral cell layer of olfactory bulb; GrO, granular cell layer of olfactory bulb; Py, pyramidal cell layer of the hippocampus; Rad, stratum radiatum of the hippocampus; MCL, molecular cell layer of cerebellum; GCL, granular cell layer of cerebellum. Scale bar in (e) = 50 μm. [file 1471-2202-11-158-S2.TIFF]
